# Selective Serotonin Reuptake Inhibitors in Human Pregnancy: To Treat or Not to Treat?

**DOI:** 10.1155/2012/698947

**Published:** 2011-12-10

**Authors:** Orna Diav-Citrin, Asher Ornoy

**Affiliations:** ^1^The Israeli Teratology Information Service, Israel Ministry of Health, P.O. Box 1176, Jerusalem 91010, Israel; ^2^The Hebrew University Hadassah Medical school, P.O. Box 12272, Jerusalem 91120, Israel

## Abstract

Selective serotonin reuptake inhibitors (SSRIs) are increasingly prescribed during pregnancy. The purpose of the present paper is to summarize and evaluate the current evidence for the risk/benefit analysis of SSRI use in human pregnancy. The literature has been inconsistent. Although most studies have not shown an increase in the overall risk of major malformations, several studies have suggested that SSRIs may be associated with a small increased risk for cardiovascular malformations. Others have noted associations between SSRIs and specific types of rare major malformations. In some studies, there appears to be a small increased risk for miscarriages, which may be associated with the underlying maternal condition. Neonatal effects have been described in up to 30% of neonates exposed to SSRIs late in pregnancy. Persistent pulmonary hypertension of the newborn has also been described with an absolute risk of <1%. The risk associated with treatment discontinuation, for example, higher frequency of relapse and increased risk of preterm delivery, should also be considered. The overall benefit of treatment seems to outweigh the potential risks.

## 1. Introduction

Selective serotonin reuptake inhibitors (SSRIs) are widely prescribed for the treatment of depression, anxiety, and other disorders. Estimates suggest that the lifetime risk for depression ranges between 10 and 25% with a peak prevalence occurring at childbearing age [1]. According to Evans et al., 9–14% of all pregnant women display signs of depression and/or have illnesses that fulfil research diagnostic criteria for depression [2]. The prevalence rates of depression during pregnancy are 7.4%, 12.8%, and 12.0%, for the first, second, and third trimesters, respectively [3]. A number of SSRIs were introduced since the 1980s, including fluoxetine, fluvoxamine, paroxetine, sertraline, citalopram, and escitalopram. They have better efficacy, tolerability, and safety compared to first-generation antidepressants, for example, tricyclic antidepressants, and are safer in overdose. They exert their effects by inhibiting the presynaptic plasma membrane serotonin transporter. The serotonin transporter mediates the reuptake of serotonin into the presynaptic terminal; neuronal uptake is the primary process by which neurotransmission via 5-hydroxytryptamine (neuronal serotonin) is terminated. Thus, treatment with an SSRI initially blocks reuptake and results in enhanced and prolonged serotonergic neurotransmission. All SSRIs share a similar mechanism of action despite having different chemical structures. The use of antidepressants and anxiolytics has shifted from the domain of psychiatry to primary care, with the discovery of more selective and safer drugs. SSRI use in pregnancy has increased over the years [4–7]. In recent years the proportion of pregnancies with SSRI exposure in the USA is 6% [6, 7]. SSRIs readily cross the human placenta [8, 9]. In spite of the widespread use of SSRIs during pregnancy and the relative extensive literature available, there are conflicting views on the safety of these drugs during pregnancy. The purpose of the present traditional literature review is to summarize and evaluate the current evidence for the risk benefit analysis of SSRI use in human pregnancy. 

## 2. Human Studies on SSRIs in Pregnancy

### 2.1. Congenital Anomalies (see Table 1)

A summary of studies on the use of SSRIs in human pregnancy is presented in Table 1. Many studies on the use of SSRIs during pregnancy have not shown an increase in the overall risk of major malformations [10–28]. Several studies have suggested that the use of SSRIs, particularly paroxetine, may be associated with a small increased risk for cardiovascular malformations [29–38]. Other studies have noted associations between SSRIs in pregnancy and specific types of relatively rare major malformations (neural tube defects, craniosynostosis, omphalocele, or right ventricular outflow tract defects) [39–41]. Some of these studies are retrospective and burdened with recall and selection bias. The largest dataset with prospective exposure information available to date is from the Swedish Medical Birth Registry. Their initial report has been negative [16]. Later, increased risk of cardiovascular defects was observed with paroxetine, predominantly of septal defects [30, 31]. Recently, new associations were noted, for cystic kidney with SSRIs, for relatively severe malformations with fluoxetine, and for any cardiovascular defect and hypospadias with paroxetine [38]. Three recently published meta-analyses on paroxetine exposure in pregnancy and cardiovascular malformations have not been consistent [42–44]. In the meta-analysis published by Bar-Oz et al. [42], first trimester paroxetine exposure was associated with a significant increase in the risk of cardiac anomalies. Women using antidepressants in pregnancy were more likely to utilize ultrasound in pregnancy and postnatal echocardiograms compared with women who did not. In this study, significantly more women receiving paroxetine used the medication for anxiety or panic disorders compared to women using other SSRIs. Detection bias was suggested as a contributing factor to the observed risk of cardiovascular malformations with paroxetine. In the meta-analysis published by O'Brien et al. [43], no increased risk of congenital malformations was associated with paroxetine. Cardiac malformation rates were similar and within population norms. In the meta-analysis published by Wurst et al. [44], there was an increased risk for combined cardiac defects and aggregated congenital defects with first trimester paroxetine use. Two opposing commentaries on this topic were recently published [45, 46]. The definition of cardiovascular malformations varied among studies, some including small septal defects, while others excluded them. The inconsistency across these studies may be explained by differences in study design, by confounding factors, for example, maternal underlying psychiatric disorder, coadministered medications, lifestyle factors (smoking, drinking), maternal BMI, and diabetes, or they may be spurious. Overall, there are over 33,000 reported pregnancy outcomes after prenatal exposure to various SSRIs. 

We have calculated the overall rate of major congenital anomalies and of cardiovascular anomalies in the published prospective studies after prenatal exposure to SSRIs, where rates were available [10–15, 20, 25, 32, 33] and found 3.8% (189/4920) and 0.9% (53/6094), respectively, both well within their baseline risk in the general population. It can be summarized that the majority of the prospective studies have not shown an increase in the overall risk of major malformations. The studies which have suggested that SSRIs may be associated with a small increased risk for malformations were particularly with paroxetine.

### 2.2. Judging the Evidence for SSRIs as Possible Causes of Major Malformations

Seven criteria for proof of human teratogenicity have been amalgamated by Shepard [51] and are presented in Table 2 and discussed as follows.

1. Many of the studies with positive findings on SSRIs in pregnancy are prescription studies, and women may not have actually taken the drugs. Exact timing of exposure during sensitive periods is often problematic, although exposure preceded the outcome.
2. There are several isolated studies with inconsistent findings of statistically significant associations. Confounding factors are often insufficiently controlled for. Many of the prospective studies are underpowered for associations between exposure and specific malformations. Many of the retrospective studies are burdened with potential biases. All studies considered here are observational. The relative risk even in positive studies is below six and the lower bound of the 95% confidence interval often close to one. The findings in regard to the type of malformations are inconsistent in the underlying studies and even in studies from the same database published at different time points.
3. In the positive studies, there was some dominance of cardiovascular malformations, septal defects in some studies, and right ventricular outflow tract obstruction defects in others. In the case of SSRIs, particularly paroxetine; however, they were non-specific. In many of the large studies, diagnosis of malformations uses classification codes and lacks careful delineation of clinical cases.
4. The fourth criterion is not relevant in the context of SSRIs. SSRIs are common exposures in pregnancy, and most of the described defects are also relatively common.
5. Animal reproductive studies in rats and rabbits administered paroxetine [52], fluoxetine [53], or sertraline [54] during organogenesis did not show a teratogenic effect.
6. There is evidence based on mouse whole-embryo studies to suggest that serotonin plays a role in cardiovascular and craniofacial development [55–58]. Paroxetine 1 *μ*M was shown to decrease serotonin-mediated proliferation of dissociated rat embryonic cardiac myocytes [59]. Rat whole-embryo culture results showed an increase in branchial bar fusion, but not cardiac malformations, after exposure to paroxetine at concentrations much higher than those achieved clinically [60]. It has been speculated that the observed malformations in vitro may be early ontogenetic indicators for infrequent cardiovascular anomalies observed in vivo. Fluoxetine was found to adversely affect cell viability and differentiation to cardiomyocytes at higher concentrations than those achieved clinically in a dose-dependent manner using mouse embryonic stem cell system [61]. SSRIs inhibit the serotonin transporter. However, developmental defects were not observed in mutant serotonin transporter knockout mice [62]. In another study, gross morphologic abnormalities were not seen in serotonin transporter knockout mice, but association was found with sudden death of the newborn mice in the first week after delivery [63]. Histologic analysis of heart sections of these mice showed that they develop cardiac fibrosis. In terms of biological plausibility, in vitro studies have been helpful in suggesting mechanistic information that adds to the plausibility of the suspected association, though in concentrations much higher than plasma concentrations in humans in clinical settings. However, as stated earlier, in vivo animal studies to date have not supported an association between in utero exposure to SSRIs and major anomalies.
7. There is evidence for placental transfer of SSRIs.

In summary, despite some troubling associations between SSRIs and major malformations, especially cardiovascular, the overall current scientific evidence has not fulfilled the criteria for proof of human teratogenicity of SSRIs. Despite having over 33,000 reported pregnancy outcomes after prenatal exposure to various SSRIs, the differences in the design of these studies and their conflicting results are confusing. One, therefore, wonders whether further well-designed epidemiologic studies, with sufficient power and good control of potential confounders will be helpful in verifying whether SSRIs are indeed associated with a small increased teratogenic risk, especially regarding cardiovascular anomalies. In our opinion, the current data do not support teratogenicity of SSRIs.

### 2.3. Miscarriage, Intrauterine Growth Restriction (IUGR), and Preterm Delivery

Most studies did not specifically focus on the impact of SSRIs on the risk of miscarriage. It was often a secondary outcome without observing a significantly increased risk. There was an increase in the miscarriage risk in two meta-analyses [64, 65]. However, in the included prospective cohort studies, crude rates were reported, and the effect of earlier gestational age at contact, which is an important factor [66], was not corrected for. In two studies that specifically focused on the risk of miscarriage, an increased risk was found with the use of antidepressants during pregnancy [67, 68], SSRIs alone, serotonin-norepinephrine reuptake inhibitors alone, and combined use of antidepressants [68]. When looking at antidepressant use by type, paroxetine alone and venlafaxine alone were associated with increased miscarriage risk. Despite an attempt to adjust for psychiatric history, the possibility of confounding by underlying psychiatric disorder could not be ruled out.

In a Finnish study, there was no increase in the rate of preterm delivery, SGA or LBW [19]. The risk of both low birth weight and preterm delivery was increased in infants who were born to mothers who had received SSRI therapy [17, 21]. Infants exposed to SSRIs had shorter gestation and lower birth weight compared to nonexposed infants [69]. The increased risk of low birth weight remained significant, even when maternal illness severity was accounted for. The adjusted OR for preterm delivery was doubled in SSRI-exposed women compared to two groups of women who had not used SSRIs during pregnancy, one with psychiatric history and another without [70]. In another study, the risk of preterm delivery was not significantly increased among SSRI users, but the risk of SGA offspring was increased among women who continued SSRI use beyond the first trimester [71]. In a study from the Quebec Pregnancy Registry, no association was found between SSRIs and the risk of SGA regardless of trimester of exposure [72]. In other studies, there was an increased risk for preterm delivery among women exposed to SSRIs in the second or third trimesters [38] or to antidepressants [73] with no increased risk for LBW or SGA. The underlying psychiatric disorder is a potential confounder in most of these studies.

In summary, associations were found in some studies between the use of SSRIs during pregnancy and risk of miscarriage, IUGR, or preterm delivery. Most of these studies are potentially confounded by the gestational age at initial contact and the underlying psychiatric disorder.

### 2.4. Neonatal Effects

Neonatal symptoms have been described initially following prenatal exposure to fluoxetine [74] and later on after exposure to paroxetine and other SSRIs [75–81]. Neonatal toxicity or discontinuation (withdrawal, abstinence) syndromes associated with SSRIs are characterized by irritability, abnormal crying, tremor, and poor neonatal adaptation including respiratory distress, tachypnoea, jitteriness, lethargy, poor tone or colour, and, rarely, convulsions. The neonatal effects have been described in up to 30% of neonates exposed to SSRIs late in pregnancy [82]. Most symptoms are mild and transient.

It can be concluded that SSRI use late in pregnancy, similar to many other psychotropic drugs, is associated with neonatal transient effects.

### 2.5. Persistent Pulmonary Hypertension of the Newborn

Some epidemiologic studies have suggested an association between maternal use of SSRIs late in pregnancy and an increased risk of persistent pulmonary hypertension of the newborn (PPHN) [38, 83, 84]. In these studies the absolute risk of PPHN was <1%. In the study which used data from the Swedish Medical Birth Register [84], the eleven infants whose mothers reported the use of SSRI in pregnancy and had PPHN survived the neonatal period. Contrary to the above, other studies, possibly underpowered, did not find such an association [26, 85]. A recent study found PPHN to be associated with mode of delivery, specifically caesarean delivery prior to the onset of labour, but not with SSRI use in the second half of pregnancy [86]. 

In summary, an absolute risk of <1% for PPHN in infants exposed to SSRIs cannot be excluded, although studies are not consistent.

### 2.6. Neurodevelopmental Effects

Most studies have focused on possible postnatal neurodevelopmental effects. Children of mothers exposed in pregnancy to fluoxetine or tricyclic antidepressants were neurodevelopmentally assessed and compared to an unexposed control group. Similar global IQ and language scores were found in the three groups [87, 88]. No significant differences in neurobehavioral scores were found between children whose mothers were taking fluoxetine during pregnancy and nonexposed children [89]. Normal development was observed in a small group of children exposed in pregnancy to citalopram and followed up to 1 year [90]. Infant developmental assessment done at 2 and 8 months of age revealed no significant differences between SSRI-exposed and unexposed infants [91]. Levels of internalizing or externalizing behaviours did not significantly differ between children prenatally exposed to SSRIs and unexposed [92, 93]. On the other hand, maternal depression and anxiety were associated with increased reports of internalizing and externalizing behaviours in their children.

Mental developmental indexes were similar in children whose mothers were diagnosed with major depressive disorder treated or untreated in pregnancy. However, children exposed to SSRIs scored lower on the psychomotor developmental indexes and the motor quality factor of the behavioural rating scale compared to unexposed children [94]. In a follow-up study using a psychomotor developmental test (Boel), abnormal test was more frequent in children prenatally exposed to antidepressants compared to unexposed [95]. In another neurobehavioral assessment study, newborns prenatally exposed to SSRIs had abnormal outcomes including increased motor activity, fewer changes in behavioural state, and abnormal sleep patterns [96]. 

Children's developmental milestones were assessed using a questionnaire at 6 and 19 months of age. Second or third trimester exposure to antidepressants was associated with later gross motor developmental milestones, though still within normal range, compared to unexposed children [97]. 

Children who had neonatal abstinence syndrome had similar mean overall developmental results compared to those who did not; however, they were more likely to have abnormal results on the social component of the Denver developmental test [98].

A recent prospective study demonstrated that SSRIs during pregnancy affect the neurobehavioral development of the human fetus [99]. Fetuses exposed to SSRIs exhibited dose-related increased motor activity and disrupted sleep. The significance of the observed changes on postnatal development is unclear.

In a recent population-based case-control study, a two-fold increased risk of autism spectrum disorders was found with prenatal exposure to SSRIs [100]. Further studies are needed to verify the suggested association. 

In summary, in most of the studies that focused on the possible neurodevelopmental effects of prenatal SSRI exposure, there is no conclusive evidence for an increased risk of adverse long-term effects.

### 2.7. Risk of Treatment Discontinuation

When evaluating the risk/benefit ratio of SSRI treatment in pregnancy, the risks associated with treatment discontinuation should also be considered. Abrupt discontinuation of psychotropic drugs in pregnancy is associated with physical and psychological adverse effects [101]. SSRI treatment discontinuation during pregnancy is associated with a higher frequency of relapse [102]. Depression is associated with an increased risk for preterm delivery [103–105]. The risk of preterm delivery increases with increasing severity of depression [106]. Treated women have lower depressive symptom scores and better functioning [105]. These risks should be a factor in the decision making in regard to treatment continuation during pregnancy.

## 3. Conclusion

Clinicians are faced with the difficult cost-benefit consideration of either making a recommendation to treat or not to treat maternal depression or anxiety with SSRIs in pregnancy. In the field of teratology, decisions on new medications during pregnancy often need to be made with insufficient human pregnancy experience on their safety. In the case of SSRIs in pregnancy, despite extensive available studies on their use, quality is more important than quantity, and data are still not conclusive. 

In summary, most studies on the use of SSRIs during pregnancy support that they are not major human teratogens. A small increased risk for cardiovascular anomalies, especially with paroxetine, cannot be excluded. There appears to be a small increased risk for miscarriages, which may be associated with the underlying maternal condition. Neonates of mothers treated with SSRIs should be closely followed up after delivery, as there is an increased risk of transient neonatal effects. There is no conclusive evidence for adverse long-term neurodevelopmental effects of prenatal SSRI exposure. Discontinuation of treatment may pose risks, for example, higher frequency of relapse and increased risk of preterm delivery. Hence, the general benefit of treatment seems to outweigh the potential small risk of untoward effects on the embryo, fetus, or neonate.

## Figures and Tables

**Table 1:** SSRIs in human pregnancy.

Study	Design	Sample size	SSRI	Results	Comments
Pastuszak et al., [10]	Prospective comparative multicentre cohort study	*n* = 128	Fluoxetine	No increase in the rate of major malformations	Small numbers

Chambers et al., [11]	Prospective comparative cohort study	*n* = 228, *n* = 101 (with physical examination)	Fluoxetine	No increase in the risk of major anomalies, higher incidence of 3 or more minor anomalies 15.5% versus 6.5%, *P* = 0.03	Physical examination by a single dysmorphologist

McElhatton et al., [12]	Prospective comparative collaborative ENTIS study	*n* = 689 antidepressants	Fluoxetine *n* = 96 Fluvoxamine *n* = 66 Paroxetine *n* = 3	No increase in the rate of congenital anomalies	Small numbers

Goldstein et al., [13]	Prospective registry, historic controls	*n* = 769	Fluoxetine	No increase in the rate of congenital anomalies	Manufacturer's data, spontaneous reports

Wilton et al., [14]	Postmarketing survey	SSRIs 187	Paroxetine *n* = 63 Fluoxetine *n* = 52 Sertraline *n* = 51 Fluvoxamine *n* = 21	No increase in the rate of congenital anomalies	Small numbers

Kulin et al., [15]	Prospective comparative multicentre cohort study	“New” SSRIs 267	Sertraline *n* = 147 Paroxetine *n* = 97 Fluvoxamine *n* = 26	No increase in the risk of major congenital anomalies	

Ericson et al.,^a^ [16]	Swedish Medical Birth Registry, initial report	SSRIs: *n* = 531	Citalopram *n* = 375 Paroxetine *n* = 122 Sertraline *n* = 34 Fluoxetine *n* = 16	No increase in the rate of congenital anomalies	Incomplete drug reporting

Unfred et al., [47]	Prospective comparative cohort study	*n* = 101	Paroxetine	Increased risk of congenital anomalies (4/96 (4.2%) 1/195 (0.5%) *P* = 0.04) no pattern	Rate of anomalies in comparison group low, is, unpublished data

Simon et al., [17]	Retrospective cohort	SSRIs: *n* = 185	Fluoxetine *n* = 129 Sertraline *n* = 32 Paroxetine *n* = 28 >1 SSRI: some	No increase in the rate of congenital anomalies; however, the rate of cardiac malformations was 2.2% in the exposed group versus 0% in unexposed	Prescription study, reliance on routinely collected clinical data, sample of live births rather than pregnancies, large number of comparisons

Hendrick et al., [18]	Review of obstetric and neonatal records	SSRIs: *n* = 138	Fluoxetine *n* = 73 Sertraline *n* = 36 Paroxetine *n* = 19 Citalopram *n* = 7 Fluvoxamine *n* = 3	No increase in the rate of congenital anomalies	Uncontrolled design, small numbers

Malm et al.,^c^ [19]	Population-based cohort study, Finnish registries	SSRI: *n* = 1398	Citalopram *n* = 554 Fluoxetine *n* = 525 Paroxetine *n* = 152 Sertraline *n* = 118 Fluvoxamine *n* = 65	No increase in the rate of congenital anomalies	Prescription study, data on dose not provided

Sivojelezova et al., [20]	Prospective comparative cohort study	*n* = 125	Citalopram	No increase in the rate of major malformations	

Wogelius et al.,^b ^ [29]	Population-based cohort study, Danish registries	SSRIs *n* = 1051	NA	Increased risk for overall anomalies (Ad RR 1.34 (95% CI 1.00–1.79) early, 1.84 (95% CI 1.25–2.71 2nd-3rd m) cardiovascular 29%	Data on specific SSRIs not provided, prescription study

Wen et al., [21]	Retrospective cohort study	SSRIs: *n* = 972	Paroxetine by 1/3	No increase in the risk of birth defects	Prescription study

Schloemp et al., [22]	Prospective comparative cohort	*n* = 119	Paroxetine	No increase in the risk of birth defects	Unpublished data

Vial et al., [23]	Prospective comparative cohort	*n* = 683	Paroxetine	No increase in the risk of birth defects	Unpublished data

Källén and Otterblad-Olausson,^a^ [30], ^a^ [31]	Swedish Medical Birth Register, updated	SSRIs: *n* = 6555	Fluoxetine *n* = 926 Citalopram *n* = 2701 Paroxetine *n* = 959 Sertraline *n* = 1906 Fluvoxamine *n* = 38 Escitalopram *n* = 72	Increased risk of cardiovascular defects with paroxetine OR 1.63 95% CI 1.05–2.53 mostly septal defects 13/20	Incomplete drug reporting, potential detection bias, multiple comparisons

Davis et al., [24]	Retrospective case control study	SSRIs: *n* = 805	Paroxetine *n* = 182	No increase in the risk of birth defects	Prescription study

Bérard et al., [48]	Retrospective nested case-control study	*n* = 1403 antidepressants	Paroxetine *n* = 542 *n *(>25 mg/d) = 143 other SSRIs *n* = 443	No increase in the rate of congenital anomalies, increased risk for overall and cardiac malformations in the high-dose (>25 mg/d) group (Ad OR 3.07 (95% CI 1.00–9.42))	Prescription study, calculation of average daily dose affected by duration in the first trimester

Alwan et al., [39]	Retrospective case-control study	Cases:*n* = 9622 Control:s *n* = 4092	SSRIs 2.4% of cases 2.1% of controls, (sertraline 0.8%, fluoxetine 0.7%, paroxetine 0.5% citalopram 0.2%)	Associations between any SSRI and craniosynostosis, paroxetine/sertraline and anencephaly, paroxetine and right ventricular outflow tract obstruction defects, omphalocele and gastroschisis—small absolute risks	Small numbers of exposed infants for individual anomalies, multiple comparisons, data on dosage unavailable, potential recall and selection biases

GSK/Cole et al., [49, 50]	Retrospective epidemiologic study	*n* = 4936 antidepressants	Paroxetine *n* = 1020	Increased risk for overall congenital anomalies for paroxetine (Ad OR 1.89 (95% CI 1.20–2.98))	Manufacturers' data from a large US insurer using data originally for a study on bupropion in pregnancy

Louik et al., [40]	Retrospective case-control study	9849 infants with defects and 5860 without	SSRIs: 2.7% of infants without malformations, 1.6–4.8% of infants with various malformations	Association between paroxetine and right ventricular outflow tract obstruction defects, clubfoot, undescended testes and NTDs, sertraline and omphalocele and septal defects—small absolute risks	Small number of exposed infants for individual anomalies, multiple comparisons, data on dosage unavailable, potential recall and selection biases

Einarson et al., [25]	Prospective comparative cohort study 8 TISes	*n* = 1174	Paroxetine	No increase in the risk of cardiac defects in the paroxetine group (0.7%) compared to controls	Spontaneously resolved cardiovascular defects not included

Diav-Citrin et al., [32]	Prospective comparative cohort study 3 TISes	SSRI: *n* = 809 1467 controls	Paroxetine *n* = 348 Fluoxetine *n* = 253	Increased Cr OR for cardiovascular anomalies after paroxetine 3.47 (95% CI 1.13–10.58) Ad OR 2.66 (95% CI 0.80–8.90), Cr OR 4.81 (95% CI 1.56–14.71) Ad OR 4.47 (95% CI 1.31–15.27) after fluoxetine	After adjustment for potential confounders OR significant only for fluoxetine and cigarette smoking of 10 or more/day, septal defects considered major anomalies even when spontaneously closed, large confidence intervals

Oberlander et al., [33]	Population-based cohort study, Canada, BC	SRIs: *n* = 2625 SRIs+BZ: *n* = 968 Controls: *n* = 107,320	SRIs: *n* = 2625 SRIs+BZ: *n* = 968 controls: *n* = 107,320	Increased risk of cardiovascular (CV) defects after combined exposure to SRI and BZ, increased risk for an ASD after SRI monotherapy, major anomalies after fluoxetine and BZ	Clinical significance of the anomaly not verified, many septal defects minor and spontaneously resolve, attempt to control for confounders

Pedersen et al.,^b^ [34]	Population-based cohort study, Danish registries	SSRIs: *n* = 1370 493,113 controls	Fluoxetine *n* = 348 Citalopram *n* = 460 Paroxetine *n* = 299 Sertraline *n* = 259 >1 SSRI *n* = 193	Increased prevalence of septal defects with sertraline (OR 3.25 (95% CI 1.21–8.75)) and citalopram (OR 2.52 (95% CI 1.04–6.10))	Prescription study, potential selection bias, underlying condition potential confounder, information on malformation from hospital registry (more sensitive to severe and visible malformations)

Wichman et al., [26]	Retrospective controlled review of medical records at the Mayo Clinic	SSRIs: *n* = 808 24,406 controls	Citalopram *n* = 122 Venlafaxine *n* = 53 Escitalopram *n* = 8 Paroxetine *n* = 134 Fluoxetine *n* = 184 Sertraline *n* = 296 >1 SSRI *n* = 11	3/808 (0.4%) had congenital heart disease after exposure to SSRIs compared with 2.05/24,406 (0.8%) without exposure to SSRIs (*P* = 0.23)	No review of SSRI exposure timing, of demographic or clinical information including use of other drugs, smoking, or alcohol use, data from physician prescription records. Small VSDs may be undetected soon after birth.

Merlob et al., [35]	Prospective comparative hospital-based study	SSRIs: *n* = 235 67,636 controls	Paroxetine *n* = 92 Fluoxetine *n* = 66 Citalopram *n* = 43 Escitalopram *n* = 13 Sertraline *n* = 8 Fluvoxamine *n* = 4 Venlafaxine *n* = 9	Non syndromic heart defects (mild) identified by echocardiography among infants with murmur on 2nd-3rd day of life RR 2.17 (95% CI 1.07–4.39)	Small sample size of exposed group, lack of data on potential confounders, ascertainment of SSRI use based on maternal report, no detection bias; all newborns with murmur examined by a pediatric cardiologist including echocardiography.

Klieger-Grossmann et al., [27]	Prospective comparative cohort study	*n* = 213	Escitalopram	No increased risk for anomalies	Unpublished data

Kornum et al.,^b^ [36]	Population-based cohort study, Danish registries, updated	SSRIs: *n* = 2064 213,712 controls	Citalopram *n* = 658 Fluoxetine *n* = 472 Sertraline *n* = 352 Paroxetine *n* = 297 Escitalopram *n* = 88 >1 SSRI *n* = 195	SSRI use associated with increased risk of overall malformations (Ad OR 1.3 (95% CI 1.1–1.6)) and cardiac (Ad OR 1.7 (95% CI 1.1–2.5)), for specific SSRIs increased risk for septal defects with sertraline (Ad OR 3.3 (95% CI 1.5–7.5))	Prescription study, potential selection bias, underlying condition potential confounder, information on malformation from hospital registry (more sensitive to severe and visible malformations)

Bakker et al., [37]	Population-based case- control study, the Netherlands	*n* = 678 with heart defects 615 controls	Paroxetine	No significantly increased risk for heart defects overall (AOR 1.5 (95% CI 0.5–4.0)) increased risk for ASD with paroxetine in T1 (AOR 5.7 (95% CI 1.4–23.7))	Small number of exposed infants for individual anomalies

Reis and Källén,^a^ [38]	Swedish Medical Birth Register, updated	SSRIs: *n* = 10, 170 1,062,190 controls	Fluoxetine *n* = 1522 Citalopram *n* = 3950 Escitalopram *n* = 153 Paroxetine *n* = 1208 Sertraline *n* = 3297 Fluvoxamine *n* = 42 Unspecified *n* = 86	Increased risk for cystic kidney (*n* = 9) with SSRIs (OR 2.39 (95% CI 1.09–4.54)), for relatively severe malformation (OR 1.29 (95% CI 1.00–1.67)) with fluoxetine, for any cardiovascular defect (OR 1.66 (95% CI 1.09–2.53) 12/24 septal defects) and for hypospadias (*n* = 9) (OR 2.45 (1.12–4.64)) with paroxetine	Prospective exposure information, largest dataset available. Incomplete drug reporting, potential detection bias, multiple comparisons, possible confounding by the underlying psychiatric condition, smoking, obesity, alcohol, folic acid, association with preexisting diabetes and hypertension

Shechtman et al., [28]	Prospective comparative cohort study	*n* = 241	Citalopram or escitalopram	No increased risk for anomalies	Unpublished data

Malm et al.,^c^ [41]	Retrospective cohort, based on Finnish population-based registries	SSRIs: *n* = 6,976	Citalopram *n* = 2,799 Fluoxetine *n* = 1,818 Paroxetine *n* = 968 Sertraline *n* = 869 Escitalopram *n* = 441 Fluvoxamine *n* = 240	Increased risk for isolated VSDs with fluoxetine (*n* = 19) (Adj OR 2.03 (95% CI 1.28–3.21)) (0.5% absolute risk increase), for right ventricular outflow tract defects with paroxetine (*n* = 3) (Adj OR 4.68 (95% CI 1.48–14.74)) (0.2% absolute risk increase), for NTDs with citalopram (*n* = 8) (Adj OR 2.46 (95% CI 1.20–5.07))	Large dataset, attempt to control for confounders (i.e., maternal age, parity, year of pregnancy, marital status, smoking, purchase of other psychiatric drugs, maternal prepregnancy diabetes). Large number of comparisons, some associations based on small numbers, drug compliance and timing of exposure in pregnancy not confirmed, septal defects considered major anomalies even when spontaneously closed

^a^From the same database of the Swedish Medical Birth Register, ^b^ from the same database of the Finnish registries, ^c^ from the same database of the Danish registries.

**Table 2:** Shepard's amalgamation of criteria for proof of human teratogenicity (Source: shepard, 1994 [51]) applied to SSRIs.

Criterion	Fulfillment by SSRIs
(1) Proven exposure to agent at critical time(s) in prenatal development.	No

(2) Consistent findings by two or more epidemiologic studies of high quality:	
(a) Control of confounding factors	
(b) Sufficient numbers	
(c) Exclusion of positive or negative bias factors	No
(d) Prospective studies, if possible	
(e) Relative risk of six or more (?)	

(3) Careful delineation of the clinical cases. A specific defect or syndrome, if present, is very helpful	No

(4) Rare environmental exposure associated with rare defect	Not applicable

(5) Teratogenicity in experimental animals	No

(6) The association should make biological sense	No

(7) Proof in an experimental system that the agent acts in an unaltered state	Evidence of placental transfer

Note: items (1), (2), and (3) or (1), (3), and (4) are essential criteria. Items (5), (6), and (7) are helpful but not essential.

## References

[1] Wisner KL, Peindle K, Hanusa BH (1993). Relationship of psychiatric illness to childbearing status: a hospital-based epidemiologic study. *Journal of Affective Disorders*.

[2] Evans J, Heron J, Francomb H, Oke S, Golding J (2001). Cohort study of depressed mood during pregnancy and after childbirth. *British Medical Journal*.

[3] Bennett HA, Einarson A, Taddio A, Koren G, Einarson TR (2004). Prevalence of depression during pregnancy: systematic review. *Obstetrics and Gynecology*.

[4] Malm H, Martikainen J, Klaukka T, Neuvonen PJ (2003). Prescription drugs during pregnancy and lactation—a Finnish register-based study. *European Journal of Clinical Pharmacology*.

[5] Egen-Lappe V, Hasford J (2004). Drug prescription in pregnancy: analysis of a large statutory sickness fund population. *European Journal of Clinical Pharmacology*.

[6] Cooper WO, Willy ME, Pont SJ, Ray WA (2007). Increasing use of antidepressants in pregnancy. *American Journal of Obstetrics and Gynecology*.

[7] Andrade SE, Raebel MA, Brown J (2008). Use of antidepressant medications during pregnancy: a multisite study. *American Journal of Obstetrics and Gynecology*.

[8] Hendrick V, Stowe ZN, Altshuler LL, Hwang S, Lee E, Haynes D (2003). Placental passage of antidepressant medications. *American Journal of Psychiatry*.

[9] Rampono J, Simmer K, Ilett KF (2009). Placental transfer of SSRI and SNRI antidepressants and effects on the neonate. *Pharmacopsychiatry*.

[10] Pastuszak A, Schick-Boschetto B, Zuber C (1993). Pregnancy outcome following first-trimester exposure to fluoxetine (Prozac). *Journal of the American Medical Association*.

[11] Chambers CD, Johnson KA, Dick LM, Felix RJ, Jones KL (1996). Birth outcomes in pregnant women taking fluoxetine. *The New England Journal of Medicine*.

[12] McElhatton PR, Garbis HM, Eléfant E (1996). The outcome of pregnancy in 689 women exposed to therapeutic doses of antidepressants. A collaborative study of the European Network of Teratology Information Services (ENTIS). *Reproductive Toxicology*.

[13] Goldstein DJ, Sundell KL, Corbin LA (1997). Birth outcomes in pregnant women taking fluoxetine. *The New England Journal of Medicine*.

[14] Wilton LV, Pearce GL, Martin RM, Mackay FJ, Mann RD (1998). The outcomes of pregnancy in women exposed to newly marketed drugs in general practice in England. *British Journal of Obstetrics and Gynaecology*.

[15] Kulin NA, Pastuszak A, Sage SR (1998). Pregnancy outcome following maternal use of the new selective serotonin reuptake inhibitors: a prospective controlled multicenter study. *Journal of the American Medical Association*.

[16] Ericson A, Källén B, Wiholm BE (1999). Delivery outcome after the use of antidepressants in early pregnancy. *European Journal of Clinical Pharmacology*.

[17] Simon GE, Cunningham ML, Davis RL (2002). Outcomes of prenatal antidepressant exposure. *American Journal of Psychiatry*.

[18] Hendrick V, Smith LM, Suri R, Hwang S, Haynes D, Altshuler L (2003). Birth outcomes after prenatal exposure to antidepressant medication. *American Journal of Obstetrics and Gynecology*.

[19] Malm H, Klaukka T, Neuvonen PJ (2005). Risks associated with selective serotonin reuptake inhibitors in pregnancy. *Obstetrics and Gynecology*.

[20] Sivojelezova A, Shuhaiber S, Sarkissian L, Einarson A, Koren G (2005). Citalopram use in pregnancy: prospective comparative evaluation of pregnancy and fetal outcome. *American Journal of Obstetrics and Gynecology*.

[21] Wen SW, Yang Q, Garner P (2006). Selective serotonin reuptake inhibitors and adverse pregnancy outcomes. *American Journal of Obstetrics and Gynecology*.

[22] Schloemp S, Paulus WE, Sterzik K, Stoz F (2006). Congenital malformations after antidepressant medication with paroxetine in early pregnancy? [abstract]. *Human Reproduction*.

[23] Vial T, Cournot MP, Bernard N (2006). Paroxetine and congenital malformations: a prospective comparative study [abstract]. *Drug Safety*.

[24] Davis RL, Rubanowice D, McPhillips H (2007). Risks of congenital malformations and perinatal events among infants exposed to antidepressant medications during pregnancy. *Pharmacoepidemiology and Drug Safety*.

[25] Einarson A, Pistelli A, DeSantis M (2008). Evaluation of the risk of congenital cardiovascular defects associated with use of paroxetine during pregnancy. *American Journal of Psychiatry*.

[26] Wichman CL, Moore KM, Lang TR, Sauver JLSt, Heise RH, Watson WJ (2009). Congenital heart disease associated with selective serotonin reuptake inhibitor use during pregnancy. *Mayo Clinic Proceedings*.

[27] Klieger-Grossmann C, Weitzner B, Einarson T, Koren G, Einarson A (2010). The safety of escitalopram in pregnancy: a prospective cohort study [abstract]. *Birth Defects Research Part A*.

[28] Shechtman S, Sabbah U, Diav-Citrin O, Ornoy A (2011). Lack of teratogenic effect of citalopram/escitalopram after in-utero exposure [abstract]. *Reproductive Toxicology*.

[29] Wogelius P, Nørgaard M, Gislum M (2006). Maternal use of selective serotonin reuptake inhibitors and risk of congenital malformations. *Epidemiology*.

[30] Källén B, Otterblad-Olausson P (2006). Antidepressant drugs during pregnancy and infant congenital heart defect. *Reproductive Toxicology*.

[31] Källén BAJ, Otterblad-Olausson P (2007). Maternal use of selective serotonin re-uptake inhibitors in early pregnancy and infant congenital malformations. *Birth Defects Research Part A*.

[32] Diav-Citrin O, Shechtman S, Weinbaum D (2008). Paroxetine and fluoxetine in pregnancy: a prospective, multicentre, controlled, observational study. *British Journal of Clinical Pharmacology*.

[33] Oberlander TF, Warburton W, Misri S, Riggs W, Aghajanian J, Hertzman C (2008). Major congenital malformations following prenatal exposure to serotonin reuptake inhibitors and benzodiazepines using population-based health data. *Birth Defects Research Part B*.

[34] Pedersen LH, Henriksen TB, Vestergaard M, Olsen J, Bech BH (2009). Selective serotonin reuptake inhibitors in pregnancy and congenital malformations: population based cohort study. *British Medical Journal*.

[35] Merlob P, Birk E, Sirota L (2009). Are selective serotonin reuptake inhibitors cardiac teratogens? Echocardiographic screening of newborns with persistent heart murmur. *Birth Defects Research Part A*.

[36] Kornum JB, Nielsen RB, Pedersen L, Mortensen PB, Nørgaard M (2010). Use of selective serotonin-reuptake inhibitors during early pregnancy and risk of congenital malformations: updated analysis. *Clinical Epidemiology*.

[37] Bakker MK, Kerstjens-Frederikse WS, Buys CH, de Walle HE, de Jong-van den Berg LT (2010). First-trimester use of paroxetine and congenital heart defects: a population-based case-control study. *Birth Defects Research Part A*.

[38] Reis M, Källén B (2010). Delivery outcome after maternal use of antidepressant drugs in pregnancy: an update using Swedish data. *Psychological Medicine*.

[39] Alwan S, Reefhuis J, Rasmussen SA, Olney RS, Friedman JM (2007). Use of selective serotonin-reuptake inhibitors in pregnancy and the risk of birth defects. *The New England Journal of Medicine*.

[40] Louik C, Lin AE, Werler MM, Hernández-Díaz S, Mitchell AA (2007). First-trimester use of selective serotonin-reuptake inhibitors and the risk of birth defects. *The New England Journal of Medicine*.

[41] Malm H, Artama M, Gissler M, Ritvanen A (2011). Selective serotonin reuptake inhibitors and risk for major congenital anomalies. *Obstetrics and Gynecology*.

[42] Bar-Oz B, Einarson T, Einarson A (2007). Paroxetine and congenital malformations: meta-analysis and consideration of potential confounding factors. *Clinical Therapeutics*.

[43] O’Brien L, Einarson TR, Sarkar M, Einarson A, Koren G (2008). Does paroxetine cause cardiac malformations?. *Journal of Obstetrics and Gynaecology Canada*.

[44] Wurst KE, Poole C, Ephross SA, Olshan AF (2010). First trimester paroxetine use and the prevalence of congenital, specifically cardiac, defects: a meta-analysis of epidemiological studies. *Birth Defects Research Part A*.

[45] Bérard A (2010). Paroxetine exposure during pregnancy and the risk of cardiac malformations: what is the evidence?. *Birth Defects Research Part A*.

[46] Scialli AR (2010). Paroxetine exposure during pregnancy and cardiac malformations. *Birth Defects Research Part A*.

[47] Unfred CL, Chambers CD, Felix R Birth outcomes among pregnant women taking paroxetine (Paxil) [abstract].

[48] Bérard A, Ramos É, Rey É, Blais L, St-André M, Oraichi D (2007). First trimester exposure to paroxetine and risk of cardiac malformations in infants: the importance of dosage. *Birth Defects Research Part B*.

[49] GlaxoSmithKline http://www.gsk-clinicalstudyregister.com/.

[50] Cole JA, Ephross SA, Cosmatos IS, Walker AM (2007). Paroxetine in the first trimester and the prevalence of congenital malformations. *Pharmacoepidemiology and Drug Safety*.

[51] Shepard TH (1994). “Proof” of human teratogenicity. *Teratology*.

[52] Baldwin JA, Davidson EJ, Pritchard AL, Ridings JE (1989). The reproductive toxicology of paroxetine. *Acta Psychiatrica Scandinavica, Supplement*.

[53] Byrd RA, Markham JK (1994). Developmental toxicology studies of fluoxetine hydrochloride administered orally to rats and rabbits. *Fundamental and Applied Toxicology*.

[54] Davies TS, Kluwe WM (1998). Preclinical toxicological evaluation of sertraline hydrochloride. *Drug and Chemical Toxicology*.

[55] Shuey DL, Sadler TW, Lauder JM (1992). Serotonin as a regulator of craniofacial morphogenesis: site specific malformations following exposure to serotonin uptake inhibitors. *Teratology*.

[56] Shuey DL, Sadler TW, Tamir H, Lauder JM (1993). Serotonin and morphogenesis. Transient expression of serotonin uptake and binding protein during craniofacial morphogenesis in the mouse. *Anatomy and Embryology*.

[57] Yavarone MS, Shuey DL, Tamir H, Sadler TW, Lauder JM (1993). Serotonin and cardiac morphogenesis in the mouse embryo. *Teratology*.

[58] Choi DS, Kellermann O, Richard S (1998). Mouse 5-HT(2B) receptor-mediated serotonin trophic functions. *Annals of the New York Academy of Sciences*.

[59] Sari Y, Zhou FC (2003). Serotonin and its transporter on proliferation of fetal heart cells. *International Journal of Developmental Neuroscience*.

[60] Sloot WN, Bowden HC, Yih TD (2009). In vitro and in vivo reproduction toxicology of 12 monoaminergic reuptake inhibitors: possible mechanisms of infrequent cardiovascular anomalies. *Reproductive Toxicology*.

[61] Kusakawa S, Yamauchi J, Miyamoto Y, Sanbe A, Tanoue A (2008). Estimation of embryotoxic effect of fluoxetine using embryonic stem cell differentiation system. *Life Sciences*.

[62] Bengel D, Murphy DL, Andrews AM (1998). Altered brain serotonin homeostasis and locomotor insensitivity to 3,4- methylenedioxymethamphetamine (“ecstasy”) in serotonin transporter-deficient mice. *Molecular Pharmacology*.

[63] Pavone LM, Spina A, Rea S (2009). Serotonin transporter gene deficiency is associated with sudden death of newborn mice through activation of TGF-*β*
_1_ signalling. *Journal of Molecular and Cellular Cardiology*.

[64] Hemels MEH, Einarson A, Koren G, Lanctôt KL, Einarson TR (2005). Antidepressant use during pregnancy and the rates of spontaneous abortions: a meta-analysis. *Annals of Pharmacotherapy*.

[65] Rahimi R, Nikfar S, Abdollahi M (2006). Pregnancy outcomes following exposure to serotonin reuptake inhibitors: a meta-analysis of clinical trials. *Reproductive Toxicology*.

[66] Schaefer C, Ornoy A, Clementi M, Meister R, Weber-Schoendorfer C (2008). Using observational cohort data for studying drug effects on pregnancy outcome-methodological considerations. *Reproductive Toxicology*.

[67] Einarson A, Choi J, Einarson TR, Koren G (2009). Rates of spontaneous and therapeutic abortions following use of antidepressants in pregnancy: results from a large prospective database. *Journal of Obstetrics and Gynaecology Canada*.

[68] Nakhai-Pour HR, Broy P, Bérard A (2010). Use of antidepressants during pregnancy and the risk of spontaneous abortion. *CMAJ*.

[69] Oberlander TF, Warburton W, Misri S, Aghajanian J, Hertzman C (2006). Neonatal outcomes after prenatal exposure to selective serotonin reuptake inhibitor antidepressants and maternal depression using population-based linked health data. *Archives of General Psychiatry*.

[70] Lund N, Pedersen LH, Henriksen TB (2009). Selective serotonin reuptake inhibitor exposure in utero and pregnancy outcomes. *Archives of Pediatrics and Adolescent Medicine*.

[71] Toh S, Mitchell AA, Louik C, Werler MM, Chambers CD, Hernández-Díaz S (2009). Antidepressant use during pregnancy and the risk of preterm delivery and fetal growth restriction. *Journal of Clinical Psychopharmacology*.

[72] Ramos É, St-André M, Bérard A (2010). Association between antidepressant use during pregnancy and infants born small for gestational age. *Canadian Journal of Psychiatry*.

[73] Einarson A, Choi J, Einarson TR, Koren G (2010). Adverse effects of antidepressant use in pregnancy: an evaluation of fetal growth and preterm birth. *Depression and Anxiety*.

[74] Spencer MJ (1993). Fluoxetine hydrochloride (Prozac) toxicity in a neonate. *Pediatrics*.

[75] Zajecka J, Tracy KA, Mitchell S (1997). Discontinuation symptoms after treatment with serotonin reuptake inhibitors: a literature review. *Journal of Clinical Psychiatry*.

[76] Stahl MM, Lindquist M, Pettersson M (1997). Withdrawal reactions with selective serotonin re-uptake inhibitors as reported to the WHO system. *European Journal of Clinical Pharmacology*.

[77] Stiskal JA, Kulin N, Koren G, Ho T, Ito S (2001). Neonatal paroxetine withdrawal syndrome. *Archives of Disease in Childhood: Fetal and Neonatal Edition*.

[78] Costei AM, Kozer E, Ho T, Ito S, Koren G (2002). Perinatal outcome following third trimester exposure to paroxetine. *Archives of Pediatrics and Adolescent Medicine*.

[79] Trenque T, Piednoir D, Frances C, Millart H, Germain ML (2002). Reports of withdrawal syndrome with the use of SSRIs: a case/non-case study in the French pharmacovigilance database. *Pharmacoepidemiology and Drug Safety*.

[80] Källén B (2004). Neonate characteristics after maternal use of antidepressants in late pregnancy. *Archives of Pediatrics and Adolescent Medicine*.

[81] Sanz EJ, De-las-Cuevas C, Kiuru A, Bate A, Edwards R (2005). Selective serotonin reuptake inhibitors in pregnant women and neonatal withdrawal syndrome: a database analysis. *The Lancet*.

[82] Levinson-Castiel R, Merlob P, Linder N, Sirota L, Klinger G (2006). Neonatal abstinence syndrome after in utero exposure to selective serotonin reuptake inhibitors in term infants. *Archives of Pediatrics and Adolescent Medicine*.

[83] Chambers CD, Hernandez-Diaz S, van Marter LJ (2006). Selective serotonin-reuptake inhibitors and risk of persistent pulmonary hypertension of the newborn. *The New England Journal of Medicine*.

[84] Källén B, Otterblad-Olausson P (2008). Maternal use of selective serotonin re-uptake inhibitors and persistent pulmonary hypertension of the newborn. *Pharmacoepidemiology and Drug Safety*.

[85] Andrade SE, McPhillips H, Loren D (2009). Antidepressant medication use and risk of persistent pulmonary hypertension of the newborn. *Pharmacoepidemiology and Drug Safety*.

[86] Wilson KL, Zelig CM, Harvey JP, Cunningham BS, Dolinsky BM, Napolitano PG (2011). Persistent pulmonary hypertension of the newborn is associated with mode of delivery and not with maternal use of selective serotonin reuptake inhibitors. *American Journal of Perinatology*.

[87] Nulman I, Rovet J, Stewart DE (1997). Neurodevelopment of children exposed in utero to antidepressant drugs. *The New England Journal of Medicine*.

[88] Nulman I, Rovet J, Stewart DE (2002). Child development following exposure to tricyclic antidepressants or fluoxetine throughout fetal life: a prospective, controlled study. *American Journal of Psychiatry*.

[89] Mattson SN, Eastvold AD, Jones KL, Harris JA, Chambers CD (1999). Neurobehavioral follow-up of children prenatally exposed to fluoxetine [abstract]. *Teratology*.

[90] Heikkinen T, Ekblad U, Kero P, Ekblad S, Laine K (2002). Citalopram in pregnancy and lactation. *Clinical Pharmacology and Therapeutics*.

[91] Oberlander TF, Misri S, Fitzgerald CE, Kostaras X, Rurak D, Riggs W (2004). Pharmacologic factors associated with transient neonatal symptoms following prenatal psychotropic medication exposure. *Journal of Clinical Psychiatry*.

[92] Misri S, Reebye P, Kendrick K (2006). Internalizing behaviors in 4-year-old children exposed in utero to psychotropic medications. *American Journal of Psychiatry*.

[93] Oberlander TF, Reebye P, Misri S, Papsdorf M, Kim J, Grunau RE (2007). Externalizing and attentional behaviors in children of depressed mothers treated with a selective serotonin reuptake inhibitor antidepressant during pregnancy. *Archives of Pediatrics and Adolescent Medicine*.

[94] Casper RC, Fleisher BE, Lee-Ancajas JC (2003). Follow-up of children of depressed mothers exposed or not exposed to antidepressant drugs during pregnancy. *Journal of Pediatrics*.

[95] Mortensen JT, Olsen J, Larsen H, Bendsen J, Obel C, Sørensen HT (2003). Psychomotor development in children exposed in utero to benzodiazepines, antidepressants, neuroleptics, and anti-epileptics. *European Journal of Epidemiology*.

[96] Zeskind PS, Stephens LE (2004). Maternal selective serotonin reuptake inhibitor use during pregnancy and newborn neurobehavior. *Pediatrics*.

[97] Pedersen LH, Henriksen TB, Olsen J (2010). Fetal exposure to antidepressants and normal milestone development at 6 and 19 months of age. *Pediatrics*.

[98] Klinger G, Frankenthal D, Merlob P (2011). Long-term outcome following selective serotonin reuptake inhibitor induced neonatal abstinence syndrome. *Journal of Perinatology*.

[99] Mulder EJH, Ververs FFT, de Heus R, Visser GHA (2011). Selective serotonin reuptake inhibitors affect neurobehavioral development in the human fetus. *Neuropsychopharmacology*.

[100] Croen LA, Grether JK, Yoshida CK, Odouli R, Hendrick V (2011). Antidepressant use during pregnancy and childhood autism spectrum disorders. *Archives of General Psychiatry*.

[101] Einarson A, Selby P, Koren G (2001). Abrupt discontinuation of psychotropic drugs during pregnancy: fear of teratogenic risk and impact of counselling. *Journal of Psychiatry and Neuroscience*.

[102] Cohen LS, Altshuler LL, Harlow BL (2006). Relapse of major depression during pregnancy in women who maintain or discontinue antidepressant treatment. *Journal of the American Medical Association*.

[103] Orr ST, James SA, Blackmore Prince C (2002). Maternal prenatal depressive symptoms and spontaneous preterm births among African-American women in Baltimore, Maryland. *American Journal of Epidemiology*.

[104] Dayan J, Creveuil C, Marks MN (2006). Prenatal depression, prenatal anxiety, and spontaneous preterm birth: a prospective cohort study among women with early and regular care. *Psychosomatic Medicine*.

[105] Wisner KL, Sit DKY, Hanusa BH (2009). Major depression and antidepressant treatment: impact on pregnancy and neonatal outcomes. *American Journal of Psychiatry*.

[106] Li D, Liu L, Odouli R (2009). Presence of depressive symptoms during early pregnancy and the risk of preterm delivery: a prospective cohort study. *Human Reproduction*.

